# The early history and emergence of molecular functions and modular scale-free network behavior

**DOI:** 10.1038/srep25058

**Published:** 2016-04-28

**Authors:** M. Fayez Aziz, Kelsey Caetano-Anollés, Gustavo Caetano-Anollés

**Affiliations:** 1 Evolutionary Bioinformatics Laboratory, Department of Crop Sciences, University of Illinois, Urbana, IL 61801, United States

## Abstract

The formation of protein structural domains requires that biochemical functions, defined by conserved amino acid sequence motifs, be embedded into a structural scaffold. Here we trace domain history onto a bipartite network of elementary functional loop sequences and domain structures defined at the fold superfamily level of SCOP classification. The resulting ‘elementary functionome’ network and its loop motif and structural domain graph projections create evolutionary ‘waterfalls’ describing the emergence of primordial functions. Waterfalls reveal how ancient loops are shared by domain structures in two initial waves of functional innovation that involve founder ‘*p*-loop’ and ‘winged helix’ domain structures. They also uncover a dynamics of modular motif embedding in domain structures that is ongoing, which transfers ‘preferential’ cooption properties of ancient loops to emerging domains. Remarkably, we find that the emergence of molecular functions induces hierarchical modularity and power law behavior in network evolution as the network of motifs and structures expand metabolic pathways and translation.

“… I saw the Aleph from every vantage point, I saw in the Aleph the earth and in the earth again the Aleph, I saw my face and viscera, I saw your face and in vertigo I wept, for my eyes had seen that secret and conjectural object whose name is usurped by men …”— Jorge Luis Borges, *The Aleph and Other Stories*.

In order to explain the structural and functional complexities of the protein world, protein domain structure must emerge from prior structural states and must fulfill the principle of spatiotemporal continuity (‘*lex continui*’ of Leibnitz) that implicitly supports evolution. We recently argued that these prior states involve the combination of dipeptides to form three-dimensional loop structures and that these non-regular structures provide the necessary flexibility to develop molecular functions and genetics^1^. Using a phylogenomic framework, we previously reconstructed evolutionary timelines of molecular accretion in which molecules acquire substructural or modular parts in their molecular makeup. These timelines are directly generated from the sequence and structure of thousands of nucleic acid molecules and millions of protein sequences encoded in hundreds of genomes. For proteins, timelines make explicit the gradual evolutionary appearance of protein domain structures and molecular functions^2^,^3^,^4^,^5^ and their combinatorial rearrangement in proteins^6^. They also allow the evolutionary tracing of chemical and biophysical properties. For example, tracing chemical mechanisms in enzymatic reactions uncovered the natural history of biocatalysis^7^. Similarly, tracing contact order (i.e. average relative distance of amino acid contacts in the tertiary structure of proteins), which is correlated to flexibility, showed that folding speed is optimized and increases in protein evolution^8^. In dynamic metabolomics networks of *Escherichia coli*, subjection to stress stochastically induces biphasic-rewiring and modularity at regular time intervals of few minutes^9^.

The formation of domains from dipeptide constituents must also involve stable intermediates that would act as scaffolds of the flexible functional loops of emerging structures smaller than the size of an average compact domain (~100 amino acid residues in length)^10^,^11^. These intermediate prior forms have been postulated to be small peptides (~25–30 residues) forming closed loops stabilized by van der Waals locks^12^,^13^. Their history has been traced back to a few prototypes that are universally present in structures and are believed to be modern determinants of molecular function^14^,^15^. In a recent study, distant evolutionary connections of these ‘elementary functional loops’ revealed patterns of motif reuse in archaeal proteins^16^. Here we map the coevolutionary history of the oldest elementary functional loop prototypes (herein referred to as loops) and protein structural domains defined at the superfamily (SF) level of the SCOP database^17^ (herein referred to as domains). We reveal remarkable patterns of emergence of molecular functions and network connectivity in the mappings of loops and domains that are likely of very ancient origin.

## Results and discussion

### Tracing the origin and evolution of molecular functions in loops of protein domains

**Figure 1:**
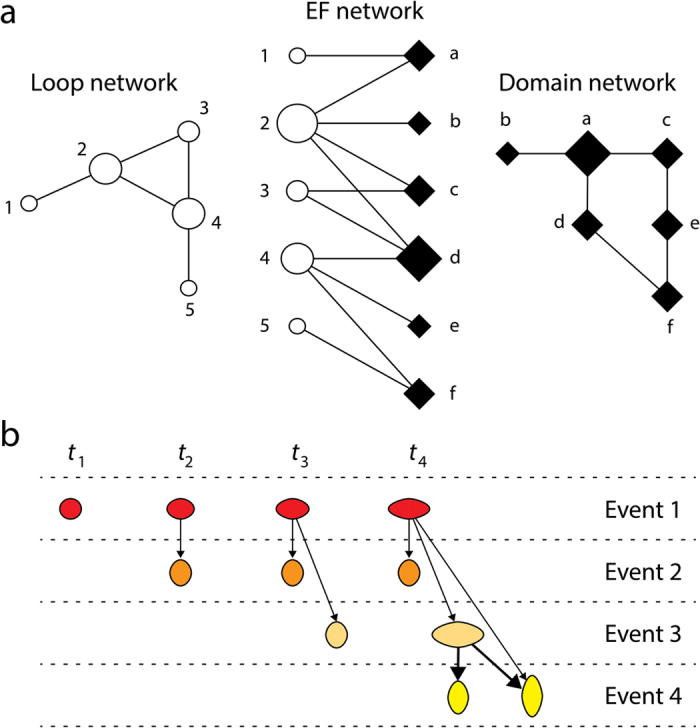
Using bipartite networks to study the evolution of elementary functionomes (EFs). (**a**) The diagrams illustrate an undirected bipartite EF network and its loop and domain network projections. Nodes are described as symbols, with size proportional to the number of links they establish. (**b**) Construction of directed ‘discrete event’ networks in waterfall format. As time progresses from left to right, events describe the progressive appearance of nodes and links. Network growth is made explicit by coloring and arranging nodes according to age (red-to-yellow and top-to-bottom), using time-induced arrows (arcs) with density proportional to their connectivity, and horizontal and vertical sizes of symbols proportional to the respective outdegree and indegree of the nodes. As the network grows with time, the transition from wide to tall symbols facilitates the visualization of the source-sink origination dynamics of recruitments. Note how the chronological accumulation of connections in the originating node (colored red) progressively increases its outdegree as connections are established with nodes of later events. This widens the symbols horizontally. In contrast, nodes appearing later in time receive connectivity from earlier nodes, increasing their indegree and hightening the vertical scale of symbols.

We mapped the evolutionary age of domain structures onto a bipartite graph-theoretic representation of domains and associated loop prototypes, which we then decomposed into its dual network projections using mathematical properties of finite graphs^18^. The strategy is described in Fig. 1a. The bipartite graph consists of two disjoint sets of entities (nodes), one describing the loops (circles) and the other the domains (rhomboids). Bipartite graphs project connectivity within each set of nodes^19^. Connections (links or undirected edges) between nodes of the two sets become the basis of connectivity between nodes in separate networks of each set of nodes. The larger the connectivity of nodes of the bimodal graph, the stronger the connectivity in the extracted single node (uni-modal) graphs. Our bipartite network yields domain and loop networks when projected into its two uni-modal network representations (Fig. 1a). Links in the domain network are established when domains embody mutual loop(s) in their makeup. Links of the loop network arise when loops combine to form active sites or are present in separate instances of the same domain.

Evolutionarily, our bipartite graph and its projections describe how prior loop forms that carried the most primordial functions rearrange to create modern repertoires of domain structures and their associated molecular functions. Hence, the bipartite network portrays the makeup and evolution of ‘elementary functionomes’ (EFs). Loop prototypes represent the oldest ancestral sequence motifs, 25–30 amino acid residues in length, which do not exist contemporarily but are represented by descendant loop sequences in modern proteomes^14^. These sequences that diverged from corresponding prototypes make up loop regions that host important functional roles. In turn, domains are considered bona fide structural, functional and evolutionary units of proteins^17^. A link between a loop and a domain in the EF bipartite network represents the embedding of that loop in the structural scaffold of the corresponding domain module. Therefore, the networks describe combinatorial (syntactic) relationships between loops and domains in which context-dependent rules (pragmatics) determine contextual rules (semantics). In simpler words, network links describe how the elementary alphabet of functions associates with the structural alphabet of domains at an evolutionary level.

The EF bipartite network *per se* and its domain and loop graph projections can only display evolutionary information if an age can be assigned to its node and link components. In other words, mapping age onto the graph representations describes how the networks grow in time (Fig. 1b). We used a structural phylogenomic analysis based on a census of domains in 749 genomes spanning all three domains of cellular life to define a timeline of structural innovation^20^. The relative age (*nd*) of each domain structure was extracted from this timeline. Calibration points of SF domains associated with microfossil, fossil and biogeochemical evidence, biomarkers, and first-appearance of clade-specific domains (integrated with molecular, physiological, paleontological and geochemical data) converted the timeline of relative ages into geological time scales^20^. Since prior elementary forms manifest into modern protein functions only when embedded into the structural scaffolds of modern domains, the ages of first-appearance of loops were directly transferred from the ages of first-appearance of associated domains or second oldest domains in multidomain proteins. Other plausible options however produced results similar to those here described.

### Capturing the early history of modern functionomes with time event ‘waterfall’ networks

**Figure 2:**
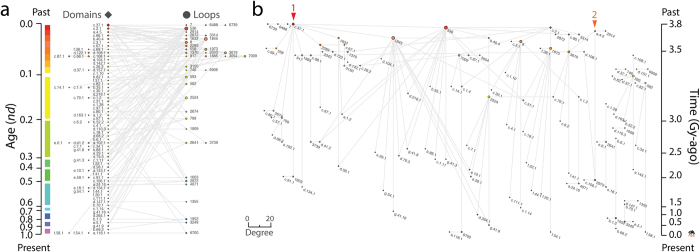
The EF network in bipartite (**a**) and waterfall (**b**) layouts. Loop and domain nodes were arranged top-down according to age (*nd*) displayed in a relative 0-to-1 scale, labeled using established SCOP nomenclature^17^ and colored according to time events (left). Ages were also time-calibrated with a molecular clock of SF domains that spans 3.8 billion years (Gy) of history using fossils and microfossils, geochemical, biochemical, and biomarker data^20^ (right). The nodes were scaled proportional to their weighted degree, i.e. the sum of the weights of all edges of the nodes. Prototype hits to structural domains in proteomes were not used to weight edges to avoid complication in interpretation of weighted network projections. Red arrowheads indicate the origin of major waves of recruitment in the time event waterfall. The horizontal expansion is dictated by VOS clustering, which elucidates formation of modules along the evolutionary timeline (see methods).

The operation of a network system can be modeled as a discrete sequence of events (network growth at temporal intervals) using Discrete Event Simulation (DES) tools^21^,^22^,^23^. DES is a form of computer-based modeling widely used to study complex behavior and how interactions between entities are affected by consecutive events. Under DES, the system does not change between events. Consequently, time flows from event to event in a discretized manner as a *step function*. Here we borrow the DES rationale to study how discrete evolutionary ‘time steps’ (intervals) materialize in the growing structure of the undirected and unweighted EF bimodal network and its uni-modal weighted projections (Fig. 1b). Events manifest time steps identified by the first appearance of domain and loop variants and their mutual links as these create novel molecular functions. We focus on the 38 most abundant profiles defining loop prototypes out of 138 identified in the 68 archaeal proteomes analyzed^15^. While the entire set of profiles hits only 6.4% of all known SF domains, the selected subset represents the most abundant and widely distributed loops, which have the oldest origin. The bipartite network of 38 loops and 82 domains resulted in a disconnected undirected graph with few small intra-connected components isolated from a large connected one. The emergent EF network had 134 edges with a *network density* (actual**/**possible number of edges) of 0.043 [134/(82 × 38)] and a *node average degree* (edges per node) of 2.23 (±0.232), i.e. EF groups had ~2 mutual connections on average. The network was well structured from a visual clustering point of view. The Visualization of Similarity (VOS) clustering method revealed 25 communities (also known as modules) with a high *modularity index* of 0.894, calculated as a standard modularity measure^24^,^25^. The event dynamics of the EF network were made evident by color coding domain and loop nodes and arranging them by age in a top-down bimodal layout following the evolutionary timeline of protein domain structures (Fig. 2a). The size of nodes was made proportional to the connectivity of nodes, measured by weighted degree, making hub-like behavior explicit in the network structure. In order to better visualize evolutionary patterns, network clusters (comprising of hubs and their constellations) were manually dissected by expanding the horizontal arrangement of the bimodal EF network with the energy-optimized Kamada-Kawai^26^ ‘optimize inside clusters only’ method (Fig. 2b). The resulting ‘waterfall’ layout makes evident the processes of functional recruitment as one travels down the events of the waterfall.

**Figure 3:**
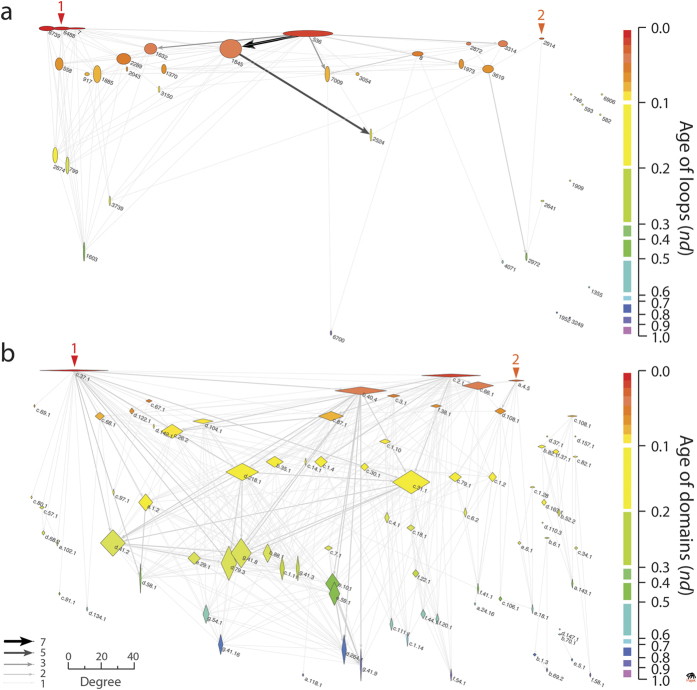
EF network projections in waterfall layout. (**a**) Loop network defined by 38 loops (ellipsoids) and arc connections (arrows) representing sharing of domain structures. (**b**) Domain network defined by 82 domains (rhomboids) and arc connections representing sharing of loop motifs. Loop and domain nodes in their uni-modal graph representations were arranged top-down according to age (*nd*) displayed in a relative 0–1 scale, labeled using established SCOP nomenclature^17^ and colored according to time events (right). The 2D scale of nodes was kept proportional to their weighted degree. In particular, the horizontal and vertical sizes of the ellipsoids (loops) and rhomboids (domains) were made proportional to the weighted outdegree and indegree, respectively, showcasing source-and-sink relationships. All weighted degree vectors were shifted by a value of 10 to avoid vanishing of 0-degree entities. The width of arcs joining the loops and domains was made proportional to the number of ‘shared domains and loops, respectively. The same criterion stands true for the grey scale of the arcs. The arcs symbolize the flow of time (random direction for contemporary nodes).

The projections of the EF graph were also visualized as waterfall networks (Fig. 3). To make evolution explicit, uni-modal network connectivity was made directional by connecting nodes with *arcs*, arrows symbolizing the flow of time from older to younger nodes. The loop and domain directed graphs were also disconnected and had 113 and 376 arcs, *network densities* of 0.080 [113/(38 × (38−1))] and 0.056 [376/(82 × (82−1))], and *node average degrees* of 3.45 (±0.551) and 5.08 (±0.534), implying ~3 domains or ~5 loops were shared on average, respectively. The uni-modal networks showed significant community structure. The loop network had 13 clusters with a modularity index of 0.608. The domain network had a comparable number of 15 clusters but with a higher modularity index of 0.886. The number of outward (outdegree) and inward (indegree) connecting arcs of nodes endowed domains and loops as ‘donors’ (sources) or ‘acceptors’ (sinks) of functional loops and domains, respectively. The horizontal and vertical scaling of nodes were made proportional to their weighted outdegree and indegree, respectively. This facilitated the visualization of hubs. The transition from wide to tall symbols along the flow of time revealed the source-sink origination dynamics of molecular functions (see below). This transition expresses the expected increase in probability of co-opting older loops and domains with time.

Two major waves of functional innovation were evident in the waterfall diagrams of the EF network and its projections (Figs 2 and 3). These waves had separate origins and involved sandwich, barrel and bundle protein domain structures. Wave 1 was the larger of the two and originated in the P-loop containing nucleoside triphosphate (NTP) hydrolase domain (c.37.1) and its uniquely connected and relatively long *p*-loop-related 7, 6488 and 6739 loops. The c.37.1 domain is a Rossmanoid α/β/α-layered domain structure that is the most ancient and popular in the timeline of domain history^2^,^27^. The *p*-loop prototype crucially enabled the nucleotide triphosphate binding functions of the P-loop hydrolase fold with its Walker A (*p*-loop) sequence motif located at the elbow, usually connecting the first β-strand of the main β-hairpin loop (or loop derivatives) that binds to di- and tri-nucleotides. The loop network shows that the ‘*p*-loop’ wave established several massive pathways of loop recruitment involving four cysteine-rich loop prototypes, the loop 536 hub, the downstream highly connected loops 1845 and 1632, and terminal loop 2524 (Fig. 3a). These cysteine-rich loops of the wave involved a strong recruitment pathway spanning ~0.5 billions of years (Gy) of history, in which the NAD(P)-binding Rossmann-fold domain (c.2.1) and the S-adenosyl-L-methionine-dependent methyltransferase domain (c.66.1) with their 3-layered α/β/α structures, and the OB-fold of the nucleic acid-binding protein domain (b.40.4) with its closed or partly-opened β-barrel structure, enabled a host of functions related to metabolism and translation. In particular, the cysteine-rich metal binding loop of loop 1845 formed a cysteine nest that coordinated Zn^2 + ^metal binding necessary for interactions with nucleic acids in 13 loop-related domains. Among these domains were the ancient OB-fold structure of b.40.4, class II aminoacyl-tRNA synthetases and biotin synthetases (d.104.1) and nucleotidyltransferase (d.218.1) domains with α/β/α-layered and sheet structures, and more derived beta and beta-prime subunits of DNA dependent RNA polymerase (e.29.1), RNA polymerase subunit (g.41.9) and prokaryotic type I DNA topoisomerase (e.10.1) domains with β-barrel and winged helix-like structures (Fig. 2b). Terminal loop 2524 of the cysteine-rich loop recruitment pathway completed the tRNA-independent cysteine biosynthetic pathway 3-3.2 Gy-ago by providing functions to the tryptophan synthase β-subunit-like PLP-dependent domain (c.79.1) of serine acetyl-transferase and *O*-acetylserine sulfhydrylase enzymes^28^. This probably enhanced cysteine availability for cysteine-rich loop recruitment and binding of Fe-S clusters, which started with the PLP-dependent transferase c.67.1 domain 3.5 Gy-ago. Finally, the loop 536 hub also linked downstream glycine and glutamate-rich loops 3314 and 7009 and the glycine-rich nucleotide-phosphate binding loop 8 that is typically embedded in β/α-barrel structures widespread in metabolism via the Rossmann c.2.1 domain structure. Loop 8 acted as hub for other downstream loops, including loops 7009 and 3619.

The second wave originated in the ‘winged helix’ DNA binding domain (a.4.5) and its uniquely connected loop 2914. The wave appeared soon after the *p*-loop wave but was much constrained in scope; part of it merged with the *p*-loop wave through loop 3619. The a.4.5 domain harbors the DNA/RNA-binding 3-helical bundle fold (a.4) structure, which is flanked by a 4-strand β-sheet. The structure exposes crucial elbows between the helix-turn-helix motifs that harbor the specificity of protein-protein and protein-RNA interactions typical of these enzymes. The winged domain structure plays central roles in transcription, providing flexibility and nucleic acid clamping capacity to RNA polymerases^29^. The structure also provides crucial surfaces for domain-domain recognition in complexes (like polymerases, ubiquitin-ligases, condensins) and other protein-protein interactions.

It is remarkable that the first two waves uncovered by the EF network and its projections involve the same primordial sandwich α/β/α-layered structures, β-barrels and helical bundle structures we identified as part of the first 54 domain families that appeared in evolution^3^. It is also remarkable that the ‘*p*-loop’ and ‘winged helix’ waves embedded the first two major gateways of enzymatic recruitment we previously identified in metabolism, the first gateway mediated by the c.37 fold and originating in the energy interconversion pathways of the purine metabolism subnetwork, and the second mediated by the a.4 fold and originating in the porphyrin and chlorophyll metabolism subnetwork and the biosynthesis of cofactors^27^,^30^,^31^. The fact that we are obtaining congruent evolutionary results with different data sets support the historical statements we here propose.

### The evolutionary dynamics of emergence of molecular functions

**Figure 4:**
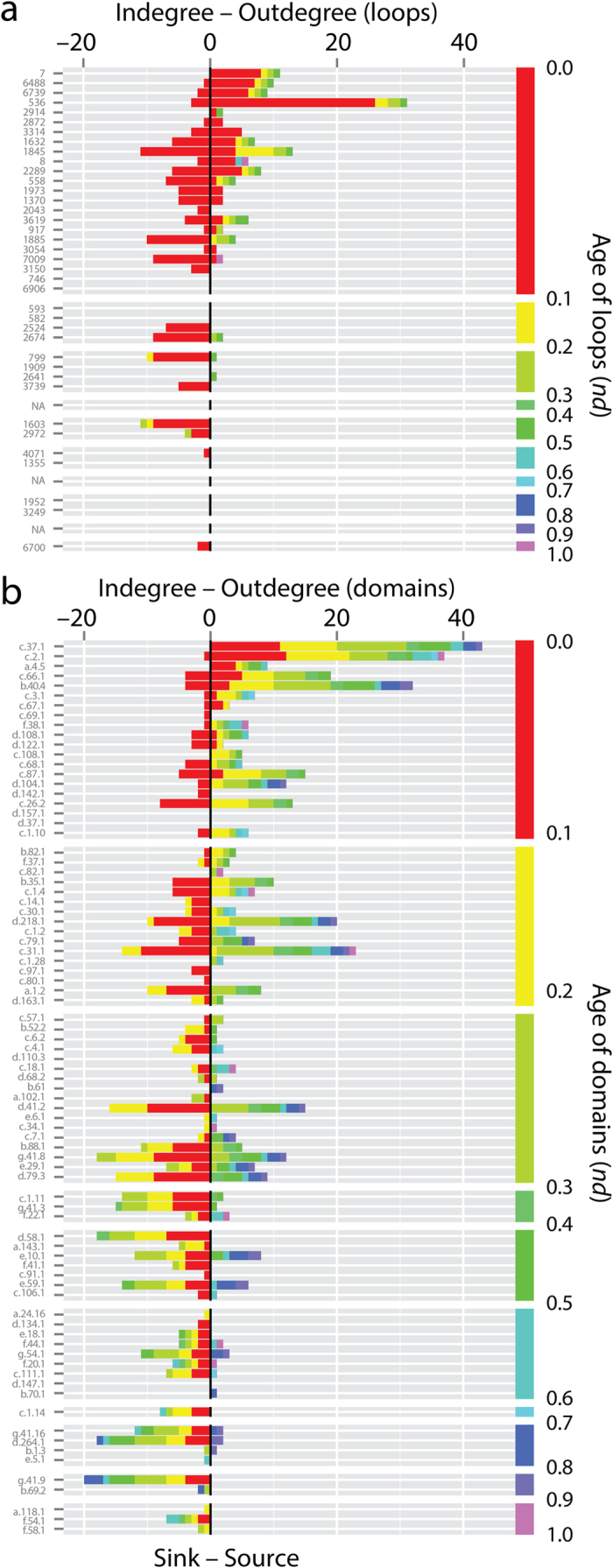
Chronological accumulation of connectivity in loop and domain networks. The stacked bar charts depict the chronological accumulation of connections (arcs) in events along the timeline of loop (**a**) and domain (**b**) innovation, which are labeled using standard SCOP nomenclature^17^. Each event corresponds to the discovery of loops and domains from one of 26 and 61 events, respectively, along a timeline that spans the origin of proteins (*nd* = 0) and the present (*nd* = 1). For visualization purposes, the timeline of events was coarse-grained into 10 age bins (colored red-to-purple). For each node in non-vacant bins, the number of connections to nodes appearing earlier (indegree) or later (outdegree) in evolution were recorded and displayed as colored stacks in the stacked bars. The charts portray sink-source relationships in the recruitment of elementary functions viewed from the perspectives of loops and domains.

The waterfall layout resulted in 61 unique events in the EF and domain networks and 26 events in the loop network along a timeline that spans the origin of proteins (*nd* = 0) and the present (*nd* = 1) (Fig. 2). The distribution and connectivity of loop and domain nodes within and across these events provides information about how dynamic, recurrent and widespread is the combinatorial recruitment process that embeds loops into domains scaffolds and generates new molecular functions. Since the loops that were studied are the most abundant and widely distributed in genomes, they are likely the oldest^15^. Indeed, the largest hubs bolstering most of the connectivity of the networks of loops and domains we sampled appeared very early in protein evolution (Figs 2 and 3). EF innovation developed during the first ~1.8 Gy of protein history (Fig. 2). However, the combinatorial recruitment process involved the entire timeline; most acceptors of loops and domains populated the *nd* = 0.0–0.1 and *nd* = 0.1–0.5 ranges, respectively. These patterns can be made quantitative by dissecting the source-sink relationships and evolutionary span of network connectivity with bar plots describing the chronological accumulation of links along the timeline (Fig. 4). Further insight can be obtained from box-and-whisker plots of accumulation of weighted indegree and outdegree (Supplementary Figure S1) and patterns of contraction or expansion of mutually-facilitated loop and domain innovation, extracted from the distributions of total degree (Supplementary Figure S2). Overwhelmingly, sink loops acted as acceptors of very ancient loops of *nd* < 0.1. In contrast, sink domains drew innovation from domains spanning the entire timeline, taking at the same time advantage of the repertoire of very ancient loops. Individual domains however co-opted a significant number of ancient domains for their functional tasks, confirming evolutionary patterns of recruitment obtained in the enzymatic analysis of metabolic networks^32^.

Connectivity patterns make explicit the evolutionary dynamics of emergence of molecular functions, falsifying some alternative hypotheses that could explain it. Historically, the creation of molecular novelty most likely involved the ligation of dipeptides and small polypeptides with limited ordered structure, followed by the formation of larger peptides harboring stable loop structures^1^,^3^, and finally the combination of loops to form defined 3D folded topologies in small protein domains^12^,^13^,^15^. While a continuum of these prior forms is expected when invoking the principle of spatiotemporal continuity, Fig. 4 reveals that the relative formation of useful loops and domains in the two waves of recruitment and innovation occurred (and is occurring) at different rates. A quick and early discovery of loops provided the raw materials for their combination in domains along the entire span of protein history. However, the generation of novel loops and domains appears ongoing and their use in combination with older domains suggests that old loops are still evolutionarily active; they are not relics tagged for extinction but evolvable forms. Thus, the fast establishment of highly conserved sequence motifs in elbow regions of loops and their combinatorial use serve to define hierarchical levels of structural complexity, which appear tightly interrelated throughout protein history. Remarkably, phylogenomic studies have also shown this same kind of dynamics materializing with domains and their combinatorial use in multidomain proteins^6^. It is noteworthy that connectivity patterns falsify evolutionary scenarios of sequential but separate build-up of loop and domain repertoires. Both loops and domains evolved in concert, but at different rates. Despite their high evolutionary conservation, results also falsify the possibility that loops are ‘molecularly canalized’ forms that resist evolutionary change. The loop prototypes arise from diverse families of sequence motifs, which express themselves in different protein structural contexts. Finally and from a historical point of view, only functionally useful prior forms would have prevailed if they provided properties that would extend the persistence of the emerging cells, including membrane stability and transport modulation, bioenergetics, and peptide-cofactor biosynthetic functions.

The loop and domain connectivity of the EF network evolved gradually from ~3 to a global average of 1.63 (±0.16) loops per domain and from 1 to a global average of 3.53 (±0.59) domains per loop (Supplementary Figure S3). Remarkably, domain connectivity decreased to 2 loops per domains in ~1 Gy of protein evolution while loops spread in domains faster, doubling in about half of that time. Thus, loop cooption was more vigorous than enhancements of economy in the number of loops in typical active sites of domains. These opposing trends suggest a frustrated dynamics of growth.

### Emergence of preferential attachment behavior typical of scale-free networks

**Figure 5:**
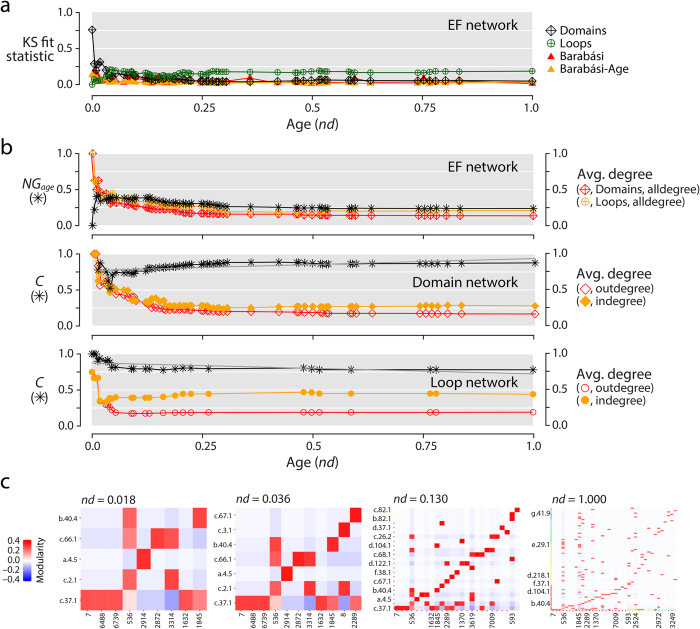
Scale-free and modular network behavior. (**a**) Transference of the scale-free property in the EF network. The KS fit statistic measures network-degree deviations from the fitted power law distribution. Lower KS indicates better fit^35^,^36^. The reference Barabási (red) and Barabási-Age (orange) curves are included for comparison. The generated scale-free network controls consider the preferential attachment probability of an old node to be proportional to its degree (Barabási) or to both its age and degree (Barabási-Age). (**b**) Modularity of growing networks. *NG* with default membership (partition) defined by age (*NG*_*age*_) was computed for the EF network. *NG*_*age*_ indicates mixing of nodes by age in an assortative (≥0) or disassortative (<0) manner across modules^39^. The average *Clustering Coefficient* (*C*) for domain and loop networks describes the averaged ratio of the triangles to the connected triples over all nodes, where the networks are simplified (undirected/unweighted)^37^,^42^,^43^. We report the coefficients of linear regression models (grey) over *C* for the domain network as 0.0022 by network size (*N*) and 0.17 by age, and those for the loop network as -0.006 by *N* and −0.15 by age. Linear regression lines are shown only by age. Normalized average degree (avg. degree) curves, computed as mean-/ max-degree of the network at an event, were included as reference controls. Separate curves were computed for the ‘alldegree’ of loop and domain portions of the EF network and for the ‘outdegree’ and ‘indegree’ of loop and domain networks. Degrees were cumulative and weighted. Scores and indices were calculated for each event of the evolving networks. Age (*nd*) is indicated in a relative 0–1 scale. (**C**) Progression of pairwise modularity in the EF network. The cells of the heatmaps represent modular strength between a loop and domain as compared to their individual connectivity with the rest of the network, scaled by network wide modularity index *NG*_*age*_ at that event^39^. The first three panels illustrate the hidden switch of power law and modularity properties between loops and domains. The last panel corresponds to the fully-grown EF network. The significant loop motifs and domain structures involved in the two major waves of functional innovation are displayed using established SCOP nomenclature^17^, ordered ascendingly and color-coded according to node age.

Networks whose dynamics follow the preferential attachment principle harbor large, highly connected hubs that attract increasingly more links in a ‘rich-get-richer’ fashion. In these highly inhomogeneous networks, which are remarkably popular in biology, the probability *P(k)* of a node being linked to *k* other nodes (i.e. the fraction of nodes with *k* links or *k*-neighbors) decays as a power law, *P(k)* ~ *k*^−γ^, without a characteristic scale. ‘Scale-free’ networks of this kind generally have exponents γ = 2.1–2.4, driving a heavy-tailed distribution in which very few nodes have high connectivity degrees^33^. For metabolic reaction networks γ = 2.2 in all organisms (e.g.^34^). In order to test if the evolving EF network and its projections had a tendency to follow the scale-free distribution, we studied the chronological accumulation of connections (links or arcs) of the growing networks and tested power law behavior with appropriate statistics (Fig. 5a, Supplementary Figures S4 and S5). Remarkably, we found that the power law and associated generative models are an emergent property of the EF network but not of its projections.

A number of statistics failed to reject power law behavior in very ancient connections of loops and most connections of domains in the EF bipartite network (Fig. 5a and Supplementary Figure S4). Thus, co-options of ancient loops by multiple domains and domains of all ages by multiple loops follow scale-free properties (Fig. 5a). The most prominent indicator of rejection was the Kolmogorov-Smirnov (KS) statistical test of power law fit^35^,^36^. Low p-values of the KS test (<0.05) and high values of the KS fit statistic (>0.10) rejected network data being drawn from the fitted power-law distribution. Similarly, the exponent of the fitted power law distribution (α), which is α > 1 when the assumption of probability of power law fit *P*(X = x^−α^) is met, only increased in degree distributions of domain nodes of the growing EF networks. These patterns are indicative of power-law decay. Finally, the log-likelihoods of the fitted power law parameters were relatively much lower than zero for most networks of the timeline, making power law distribution less likely. We found the degree distributions to be discontinuous in all of these networks.

The statistical analyses of the timeline of growing EF networks therefore reveal a surprising property of power law emergence. The early-evolved loop ‘prior form’ component of the bipartite network transfers power law behavior to the domain component as molecular functions develop in protein evolution (Fig. 5a; Supplementary Figure S4). *Log* based linear regression models overlapping with power law curves show that the coefficient of power law decay γ for the domain portion of the EF network increases with time and reaches a limit of 1.8, which is somehow lower than γ reported for metabolic networks^37^. For the loop portion, however, γ starts with ~2, but then quickly plummets to ~1 quite early in protein evolution (*nd* ~ 0.2). The coefficient of determination (R^2^) of ~80% (but never < 50%) supports the linear models. Remarkably, loop (0.407 ± 0.028) and domain (0.656 ± 0.026) network projections maintain a lower average γ < 1, consistent with statistics that reject power law behavior of these network projections. The unexpected result of power law transfer from loop to domain components may be indicative of a global scaling phenomenon in the biphasic emergence of biological modules^38^, which we now explain.

### Hierarchical modularity and the rise of primordial functions

Networks are modular when they embed communities (modules) of nodes that connect preferentially to each other within bounds of a community^39^. Modularity counteracts the scale-free property of biological networks by equalizing the degree distribution of nodes in communities^34^,^40^,^41^. However, both properties can be reconciled when modules are integrated hierarchically^37^. Modularity is primarily measured with the *average clustering coefficient* (*C*), the ratio of triangles (graph cycles of length 3) to the connected triples in the graph, averaged over all nodes, ignoring the direction and weights of the edges^37^,^42^,^43^. Since bipartite networks have no triangles, we studied the modular organization of the EF network through *C* of its projections (Fig. 5b; Supplementary Figure S6). The domain and loop networks exhibit *C* of ~0.83, significantly higher than ~0.6 reported for metabolic networks^37^,^41^,^44^. Elevated *C* of EF network projections suggests various densely intra-connected modules of loop motifs and domain structures integrated by few inter-modular links. The EF network must therefore embody a highly consolidated modular structure.

*C* of scale-free models sharply declines with network size N as N^−0.75^^45^, contrary to being independent of N if the networks are highly modular^37^. For the domain and loop networks, *C* regressed with N as N^0.0022^ and N^−0.006^, and with age *nd* of the networks as *nd*^0.17^ and *nd*^−0.15^ (Fig. 5b; Supplementary Figure S6), respectively, confirming the modular structure of the evolving networks. As expected, *C* of strictly power-law (Barabási) reference controls were zero^46^. The timeline of growing domain and loop networks revealed trends of modularity and scale-free behavior that were anticorrelated. For example, the *C* of the domain network shows an initial decline and then a rise in modularity (starts with 0.8, drops to 0.5 at *nd*~0.063, followed by growth to 0.875). This trend matches the KS fit indegree statistic that eventually rejects power law behavior (starts with 0.3, drops to 0.125 and then rises to 0.375) (Fig. 5b; Supplementary Figures S4 and S6). Since the counteracting trends of modularity and preferential attachment of the domain and loop networks must impact the emergent scale-free behavior of the EF bipartite network, our findings lead to a noteworthy conjecture. Transfer of scale-free properties from loop to domain components of the functionome involve the generation of modular and hierarchical structure of interacting loop motifs and domain structures.

To test this conjecture, we analyzed the dynamics of three additional measures of network modularity along the timeline of growing networks. The Newman-Girvan (*NG*) algorithm iteratively calculates edge ‘betweenness’, i.e. the maximum number of shortest paths running through an edge, while systematically removing edges with high measures of inter-community centrality^39^. Removal uncovers the community structure of a network measured by the *NG* index. *NG* partitioned by age (*NG*_*age*_) ranges from −1 to 1. Positive values indicate modular connectivity within events while negative values indicate connectivity across them. *NG* partitioned by VOS (*NG*_*vos*_) describes the cohesiveness of VOS divisions^24^,^25^. Finally, the Fast Greedy Community (*FGC*) detection algorithm provides a hierarchical perspective of agglomerative community structure^47^. Remarkably, *NG*_*vos*_ and *FGC* indices produced similar patterns of growth of community cohesiveness and agglomerative structure with age for all growing networks. Conversely, *NG*_*age*_ dissected divergent dynamic behaviors in the EF network and its projections. The EF network revealed an age-linked modular structure along the timeline, with an initial spike of *NG*_*age*_ ~0.5 (*nd*~0) followed by a gradual decrease to ~0.25 (*nd*~0.37). The domain and loop projections developed instead an age-independent modular structure, with initial *NG*_*age*_ of about −0.5 and −0.25 (*nd*~0), respectively, which quickly flattened towards 0 (*nd*~0.1) (Fig. 5b; Supplementary Figure S6). In these networks, communities of nodes with various ages are indicative of modularity patterns of recruitment. It thus appears that during functionome emergence, loop motifs and domain structures of the EF network were tightly coupled by age. This trend diminished as network agglomerative modularity matured and existing forms engaged in widespread recruitment of emerging constructs throughout the timeline. The recruitment trend is demonstrated in the pairwise *NG*_*age*_ heat maps of the EF network by a red sigmoidal signal during early events (first three panels) diffusing into a pervasive (red pixelated) pattern (Fig. 5c; Supplementary Video 1). Evidently, these patterns were induced by a parallel development of hierarchy that encouraged modules to cluster within modules in a fashion not distinct from the scale-free organization of modules in metabolic networks^37^ (Supplementary Figure S7; Supplementary Video 2). In contrast, the growth of loop and domain networks was initially driven by recruitment, a trend that was quickly but moderately counteracted by age-bound modularity. Thus, the transfer of scale-free properties from loop motifs to domain structures involves a hidden switch to modular and hierarchical structure, which occurred ~3.4 Gy ago. Remarkably, the timing of this switch coincides with the early development of genetic code specificity in emerging aminoacyl-tRNA synthetases and the ribosome^1^ that was facilitated by the OB-fold structure (Fig. 2).

## Conclusions

Tracing evolutionary age onto growing EF networks and their projections uncovered two clear waves of functional innovation that involved ancient loops and founder ‘*p*-loop’ and ‘winged helix’ domain structures. We find that the dynamics of recruitment of loop motifs by domain structures is ongoing and highly modular. The emergence of molecular functions showed properties of hierarchical modularity and emergent power law behavior. Modules in the EF network behaved as integrated communities of interacting structural parts defining classes of molecular functions. Our analyses suggest that module emergence occurred through a biphasic process of diversification^38^. In a first phase, the parts of the emerging functionome (loop motifs and domain structures) associated massively through processes of recruitment. Their linkage was weak. As motifs and structures diversified, they engaged in competition and were selected for functional performance. Useful emerging interactions constrained their associations. This caused tightly linked parts to self-organize into modules expressing as closely-knit communities of interactions. In a second prolonged phase, variants of the modules evolved and now became parts of a new generative cycle of higher-level organization governed by scale-free module recruitment that is still ongoing in biology. We have already shown that biphasic patterns manifest in the size, dipeptide makeup, and loop-mediated flexibilility of proteins^1^,^8^, which is likely linked to their intrinsic disorder. Thus, the generation of biphasic patterns of change may be a general phenomenon in network biology.

## Methods

### Experimental Design

#### Domain and loop prototype data

Domain structures were defined at SF level according to SCOP version 1.75^17^. The relative ages of first appearances of domains, calculated as node distance (*nd*) values from phylogenomic trees, were obtained from a previously published timeline of protein domain evolution^20^. The timeline was derived directly from a phylogeny describing the evolution of 1,730 SF domains reconstructed from a census of domain structure in 749 genomes of 52 archaeal, 478 bacterial and 219 eukaryal organisms (dataset A749). A calibrated molecular clock of SF structures (t = −3.831*nd* + 3.628) was used to calculate geological age in Gy^20^. Loop prototypes were previously identified computationally in the complete genome sequences of 68 archaeal organisms by iteratively deriving sequence profiles with a scoring function that weighs profile positions according to information content followed by hierarchical clustering^15^. The strongest 43 loops out of 138 clustered profiles were selected and mapped against non-redundant domains of the SCOP 1.75 database. From these 43 loops, 38 functionally annotated loops were used to establish evolutionary connections with domains responsible for molecular functions^15^. These loops represent prototypes showing at least 2 hits to structural domains at *E*-value < 1 and coverage of SFs in proteomes of more than 5%. Sequence logos and additional loop information can be found elsewhere^15^. Since loops are embedded in domain structure and both loops and domains describe functional and structural abstractions, the age of domains can be directly transferred to loops. We used two likely schemes to do this: (i) the age of the loop is the age of the most ancient associated domains, or (ii) the age of the loop is the age of the most recent of the most ancient couple of associated domains (or the age of the single associated domain). The mappings consider the age of a loop as either the age of the first structural scaffold or the age when the loop function is first transferred between structural scaffolds, respectively. Since both schemes provide similar mappings, we only show mappings derived using the second more conservative scheme.

### Network visualization and analysis

Networks were visualized and analyzed using Pajek^48^ and R’s *igraph* package^49^. Community-based layouts of the networks were generated using the Visualization of Similarity (VOS) clustering method^24^,^25^. Network properties were analyzed with graphing code constructs and packages of R^50^,^51^. A detailed description of data files, partitions and functions used to analyze network data, produce charts and graphs, compute power law statistics and modularity indices, and construct waterfall diagrams can be found in the Supplementary Materials and Methods file.

### Statistical Analysis

#### Power law network behavior

Scale free network behavior was studied using *P(k)* vs. *k* (i.e. probability of having *k-*neighbors vs. *k*) and *log-log* (i.e. log of *P(k)* vs. log of *k*) mappings, with linear regression models to derive γ of the power law and the determination coefficient (R^2^). γ is the negative of the slope of the log linear model. Higher γ indicates increased tendency towards preferential attachment. R^2^ describes the percentage of the data fitting the linear model. When both γ and R^2^ are high, scale free behavior should be considered strongly supported. Other power law statistics included: (i) the exponent of the fitted power law distribution (α), which assumes P(*X* = *x*) is proportional to x^−α^; (ii) KS fit statistic, which compares the fitted distribution with the input degree vector; and (iii) the KS *p*-value, with the null hypothesis of data being drawn from the power law distribution^35^,^36^. Higher α, smaller KS fit scores, and larger KS *p*-values (≥0.05) suggest better fit to power law distributions. We also determined the maximum log likelihood of the fitted parameters and if the power law fit pattern was continuous. Reference networks were created using ‘Barabási’ 52 methods of R’s *igraph* package^49^ to simulate power law and extended age-dependent graph models for the corresponding networks.

#### Network modularity

We studied modularity with six indices: (i) The *VOS Quality index* (*VQ*), was generated by the Pajek layout algorithm that takes into account values (weights) of lines (edges/arcs) as similarities. Similar communities were iteratively drawn closer to each other and the quality index of the final layout with least crossings and closest clusters was given. *VQ* is then calculated as ∑_i = 1→c, j = i + 1→c_ (e_ij_ − a_i_^2^), where c is the number of communities. e_ij_ is the fraction of edges with one node v in community i and the other w in community j, given as ∑_vw_ (A_vw_/2 m) with 1_v ϵ ci_, 1_w ϵ cj_, where m is the sum of weights in the graph and A_vw_ = the weighted value or 0 meaning presence or absence of edge between nodes v and w, in the adjacency matrix A of the network. Finally, a_i_ is the fraction of weighted k neighbors that are attached to nodes in community i, i.e k_i_/2 m^24^,^25^; (ii) The *Clustering Ratio* (*C-ratio*) considers the ratio of the number of node clusters to the count of the connected node set; (iii) The average *Clustering Coefficient* (*C*) describes the mean of the ratio of the triangles to the connected triples for all nodes in the simplified (undirected/unweighted) network^37^,^42^,^43^. *C* is only and strictly meaningful for unipartite graphs^46^. We report coefficients of linear regression over *C* for domain and loop network projections; (iv) The *Fast Greedy Community* (*FGC*) hierarchical agglomeration algorithm detects community structure with linear running time O(m d logn) ~ O(n log^2^n), with m edges, n nodes, and d, the depth of the dendrogram describing the community structure^47^; and (v and vi) The *Newman-Girvan* algorithm index (*NG*), computed with default partitions defined by age (*NG*_*age*_) and VOS clustering (*NG*_*vos*_). *NG* calculates the modularity of a network with respect to some division (partition) and measures how good the division is in separating the different node types from each other, to indicate assortative (positive) or disassortative (negative) mixing across modules^39^. *NG* equals 1/(2 m)∑_ij_(A_ij_−1/(2 m)k_i_k_j_*∆(c_i_,c_j_)), where m is the total weights in the graph, A_ij_ are weighted entries in the adjacency matrix, k_i_, k_j_ and c_i_, c_j_ are respectively the weighted degrees and the components (numeric partitions) of nodes i and j, and finally, ∆(x,y) is 1 if x = y and 0 otherwise^39^. We also computed *NG* for two additional types of membership, *FGC* and *Walk Trap Community* (*WTC*) detection algorithm. *WTC* is similar to *FGC* but computes communities using random walks^52^. *VQ, C-ratio, C* and *FGC* range from 0 to 1, while the *NG* indices range from −1 to 1. Higher indices represent strong network modularity at a particular event. Heatmaps were customized from scaled modularity matrices with elements given as (A_ij_−k_i_k_j_/(2 m))M_*nd*_, where A_ij_, k_i_, k_j_ and m are as defined for *NG*^39^ and M_*nd*_ is network’s modularity index at event *nd*. Dendrograms were calculated from squared Euclidean distance matrices indicating dissimilarities between the cluster means^53^. The distance (or dissimilarity) matrices were hierarchically clustered with the Ward’s minimum variance method aiming at finding compact, spherical clusters^55^.

## Additional Information

**How to cite this article**: Aziz, M. F. *et al*. The early history and emergence of molecular functions and modular scale-free network behavior. *Sci. Rep.* **6**, 25058; doi: 10.1038/srep25058 (2016).

## Supplementary Material

Supplementary Information

Supplementary Video 1

Supplementary Video 2
