# Supplementary material for: The early history and emergence of molecular functions and modular scale-free network behavior
Source: Sci Rep. 2016 Apr 28;6:25058. doi: 10.1038/srep25058 (PMC4848518; doi:10.1038/srep25058)
Supplement: Supplementary Information [file srep25058-s1.pdf]

SCIENTIFIC REPORTS

The early history and emergence of molecular functions and modular scale-free network behavior

M. Fayez Aziz, Kelsey Caetano-Anollés and Gustavo Caetano-Anollés

Evolutionary Bioinformatics Laboratory, Department of Crop Sciences, University of Illinois, Urbana, IL 61801, United States. Correspondence and requests for materials should be addressed to G.C.-A (e-mail: gca@illinois.edu)

Supplementary Information

Supplementary Figures

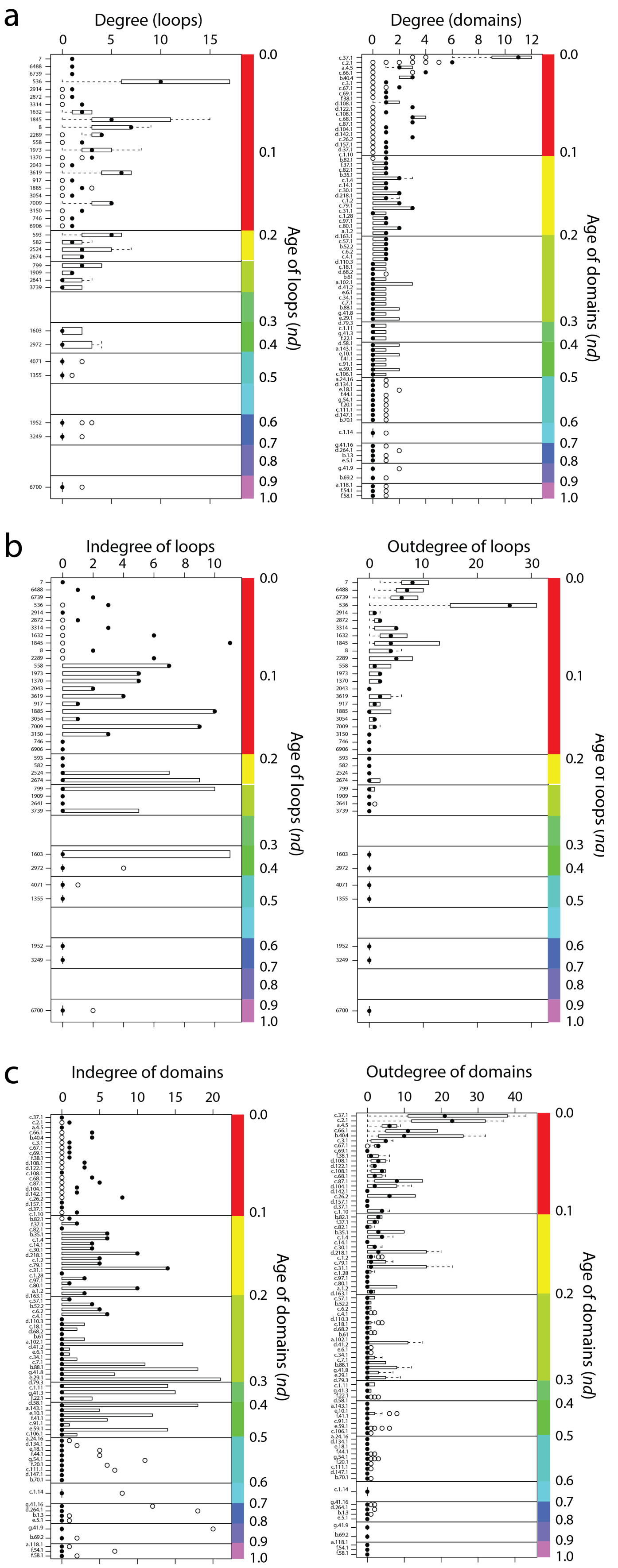

Supplementary Figure S1. Boxplots of the EF bipartite network (A) and its loop (B) and the domain (C) network projections. The Tukey boxplots show boxes (first and third quartiles bracketing the median), whiskers (values within ±1.5 × inner quartile range), and outliers. The plots describe the span of connectivity given as expansion in degree of each node along the events of evolutionary timeline. They provide a view of chronological accumulation of connections (in the form of links or arcs) with time. For the EF network, connectivity of loop and domain portions is given separately. For the projected loop and domain networks, arc connectivity is given as node indegree and outdegree and plotted separately. Each event corresponds to the discovery of loops and domains, labeled using standard SCOP nomenclature[17], from one of 26 and 61 events, respectively, along a timeline, which are labeled using relative 0-to-1 scale on the right of the plots. The timeline of events was coarse-grained into 10 age bins (colored red-to-purple).

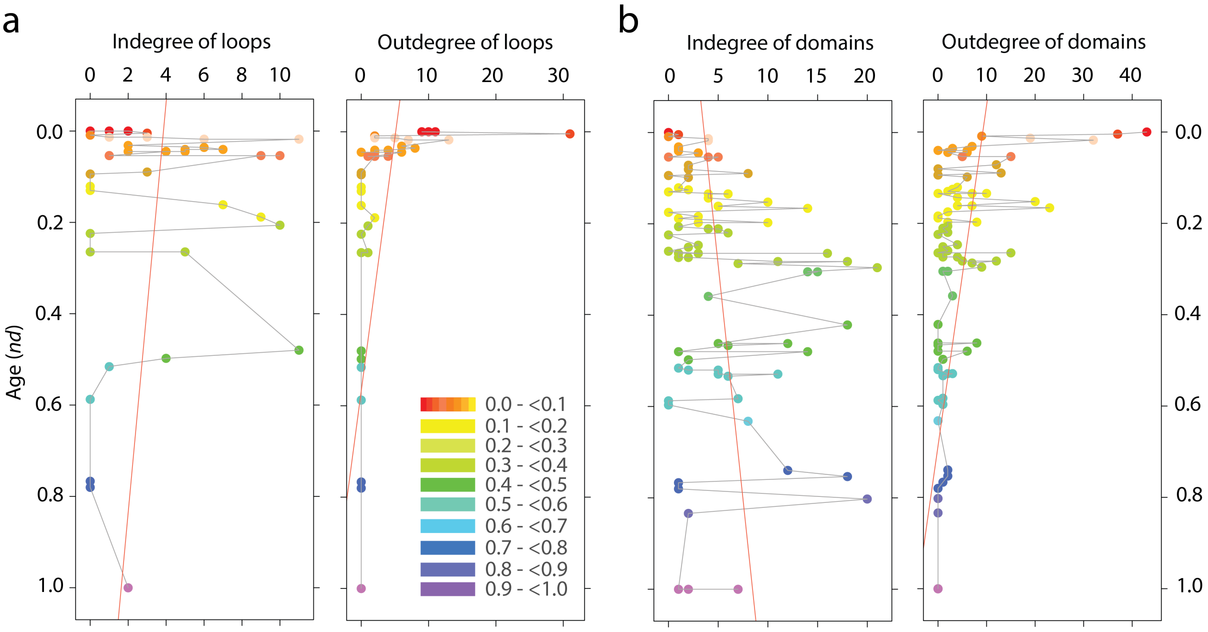

Supplementary Figure S2. Connectivity of events along the timeline in the projections of the EF network, the loop (A) and the domain (B) networks. Data points describe indegree and outdegree of nodes of different ages of networks analyzed at present time (*nd* = 1). Symbols were color-coded according to node age. Linear regression lines (red) show that the only trend of growth of connectivity with age is the embedding of old loops in emergent domains to make new molecular functions (indegree of panel **B**).

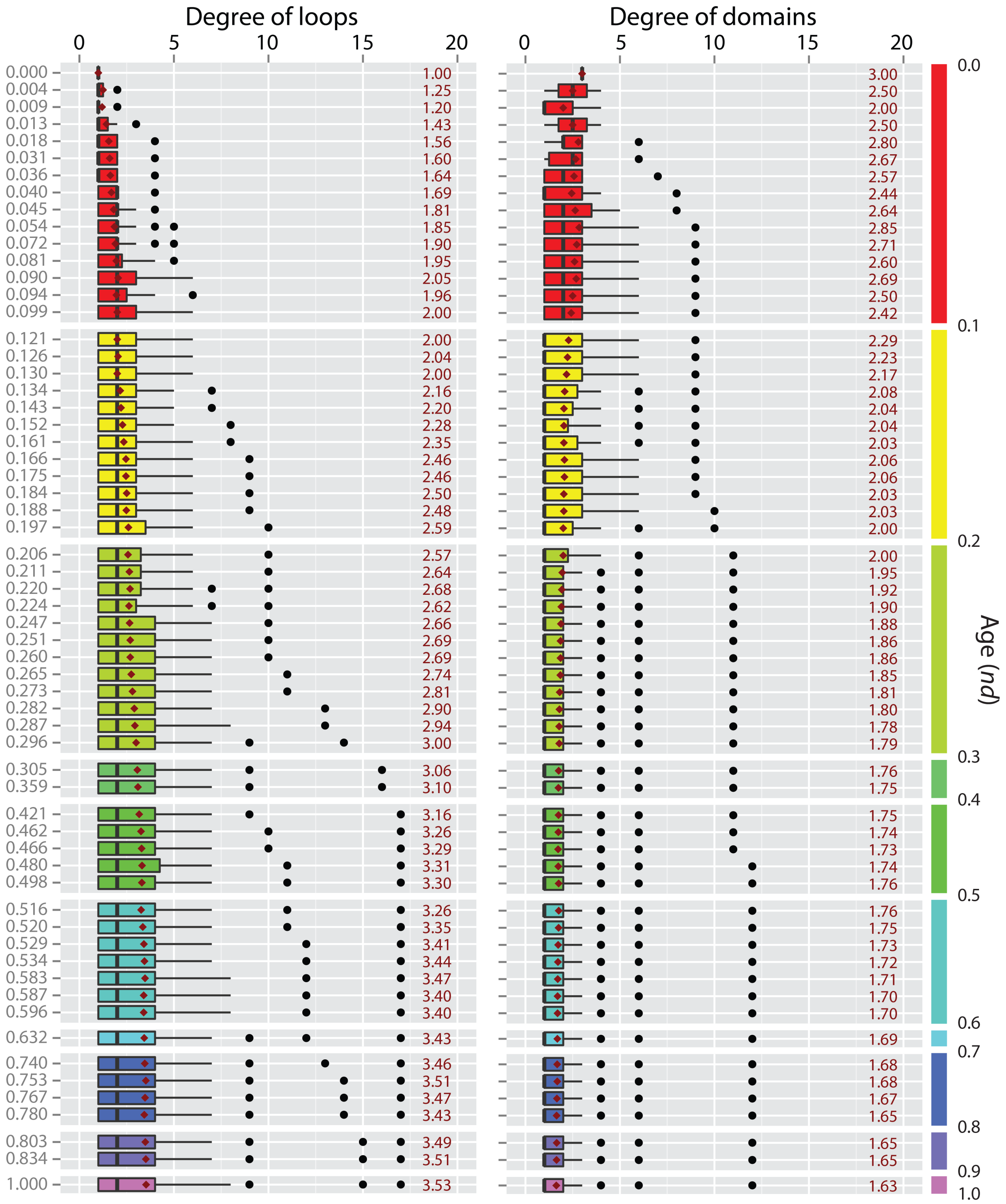

Supplementary Figure S3. Measure of central tendency and dispersion in loop and domain components of the EF network. The accumulation (and dispersion) of node degrees of the bipartite network components with age (*nd* value) was captured through Tukey boxplots with boxes (first and third quartiles bracketing the median), whiskers (values within ±1.5 × inner quartile range), and outliers. Each boxplot describes the span of cumulative connectivity along events of the evolutionary timeline by providing visualization of chronological accumulation of undirected connections with time. Average values of connectivity of loops and domains were mapped using red diamonds and reported with two digits of significance. Each one of the aggregate 61 events along the timeline are labeled with their relative age in a 0-to-1 scale. The timeline of events was also coarse-grained into 10 age bins (colored red-to-purple).

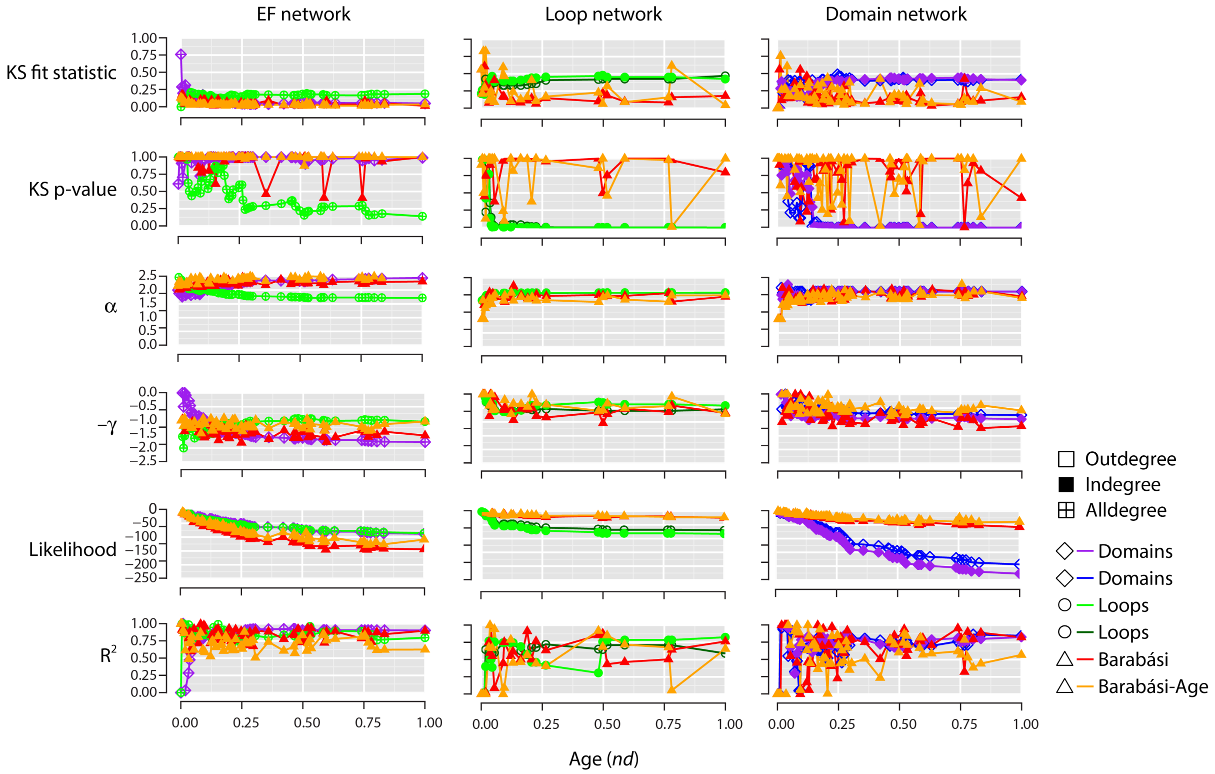

Supplementary Figure S4. Statistical descriptors of power law behavior for the EF bipartite network and its loop and domain network projections. Six differential indicators of preferential attachment were plotted for each network. Two reference curves, Barabási (red) and Barabási-Age (orange), were included for comparison. Barabási specifies the probability of preference of an old node as $P_i \sim k_i^{\alpha}$ while Barabási-Age confers heavier power law properties to older (with smaller *nd*) nodes using $P_i \sim (k_i^{\alpha})(l_i^{\beta})$. Here, k_i is the indegree of node i in the current event; α is the preferential attachment exponent, with default value one, i.e. linear preferential attachment; l_i is the age of node i, i.e. the number of events elapsed since the node was added, with maximum number measured by the 'aging.bin' parameter; β is the exponent of aging, kept at 1 for linear increases in probability of preference of an older node (with high l_i). Separate curves were computed for the 'alldegree' of loop and domain portions of the EF network and for 'outdegree' and 'indegree' of projected loop and domain networks. Degrees were cumulative and weighted. The six power law indices include: (**i**) the KS fit statistic that scores deviation of input degree data vector from the fitted power law distribution; smaller scores denote better fit; (**ii**) the KS p-value less than α=0.05 indicate rejection of the null hypothesis of degree data being drawn from the fitted power-law distribution[35,36]; (**iii**) the exponent of the fitted power-law distribution (α); (**iv**) the slope of power-law linear regression model (-γ); (**v**) the log-likelihood of the fitted parameters (likelihood); and the (**vi**) coefficient of determination (R^2) indicating confidence in the linear model through percentage of degree data explained by the regression line. Higher α, lower -γ and closer to zero likelihood correspond to power law behavior. Statistics were calculated for each event of the growing networks. Age (*nd*) is indicated on a relative 0-to-1 scale.

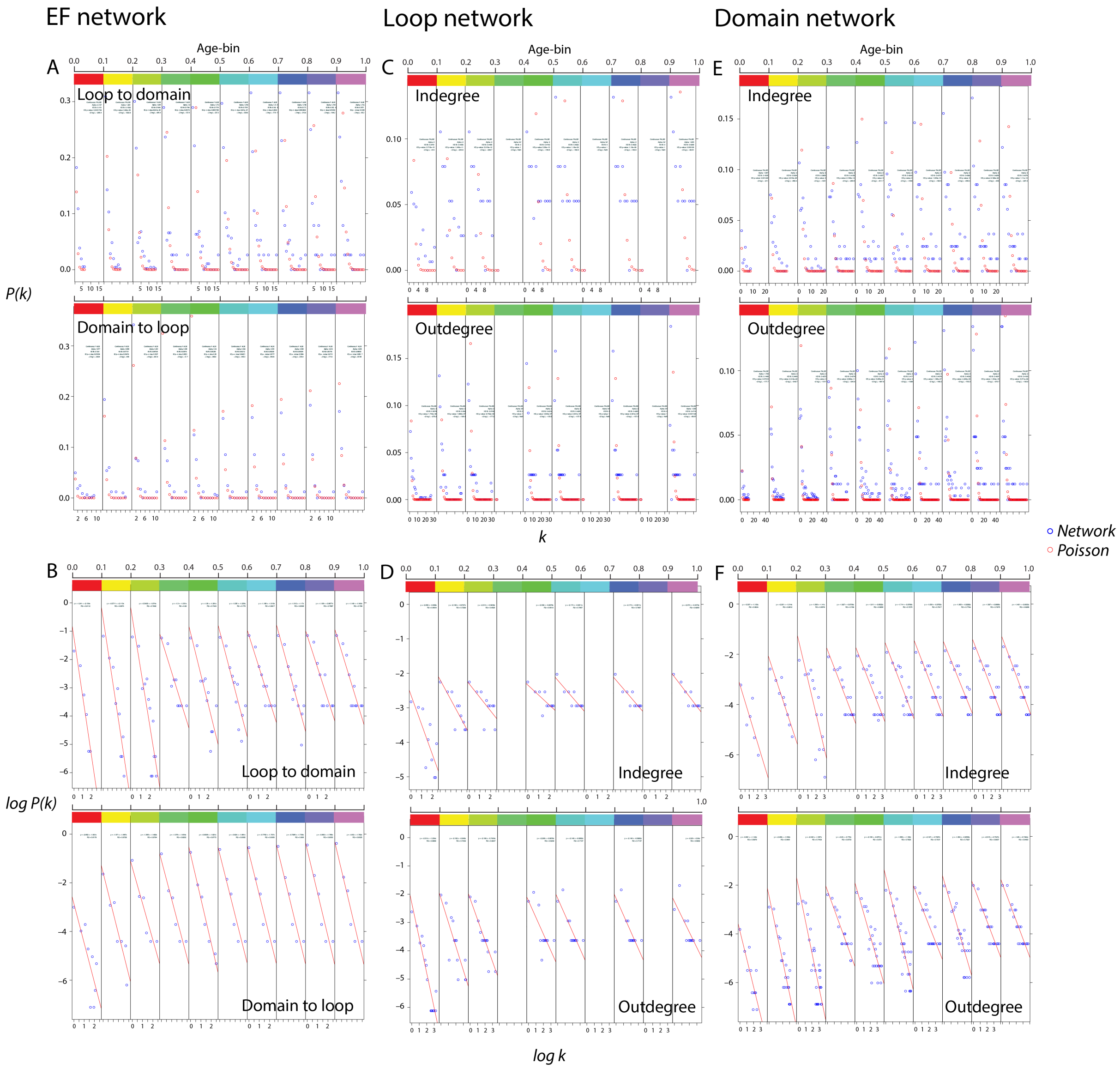

Supplementary Figure S5. ***P(k)* vs. *k* and *log-log* plots of the EF bipartite network (A,B) and its loop (C,D) and domain (E,F) network projections**. The *P(k)* vs. *k* graphs (blue) describe the relation of the range of cumulative weighted degree (1 – maximum), *k*, and the probability of occurrence of degree values, *P(k)*. The *P(k)* vs. *k* plots include a control *Poisson* distribution (red) and describe four power-law statistical descriptors: KS fit statistic, KS p-value, α and likelihood (-2 log L)[35,36]. *Log-log* graphs show a scatter-plot of natural log of *k* against *P(k)* (blue). A linear regression line (red) over the *log-log* data points is included and the slope -γ and coefficient of determination R^2 of the linear model are displayed in the inset. For the EF network, separate plots were generated for the loop and domain portions. For the projected domain and loop networks, indegree and outdegree *P(k)* vs. *k* and *log-log* graphs were plotted separately. Cumulative weighted degree was coarse-grained into 10 age-bins (colored red-to-purple), corresponding to the discovery of loops and domains from one of 26 and 61 events, respectively. Notice that few age-bins in loop network are empty. The timeline is displayed in a relative 0-to-1 scale on the top of the plots.

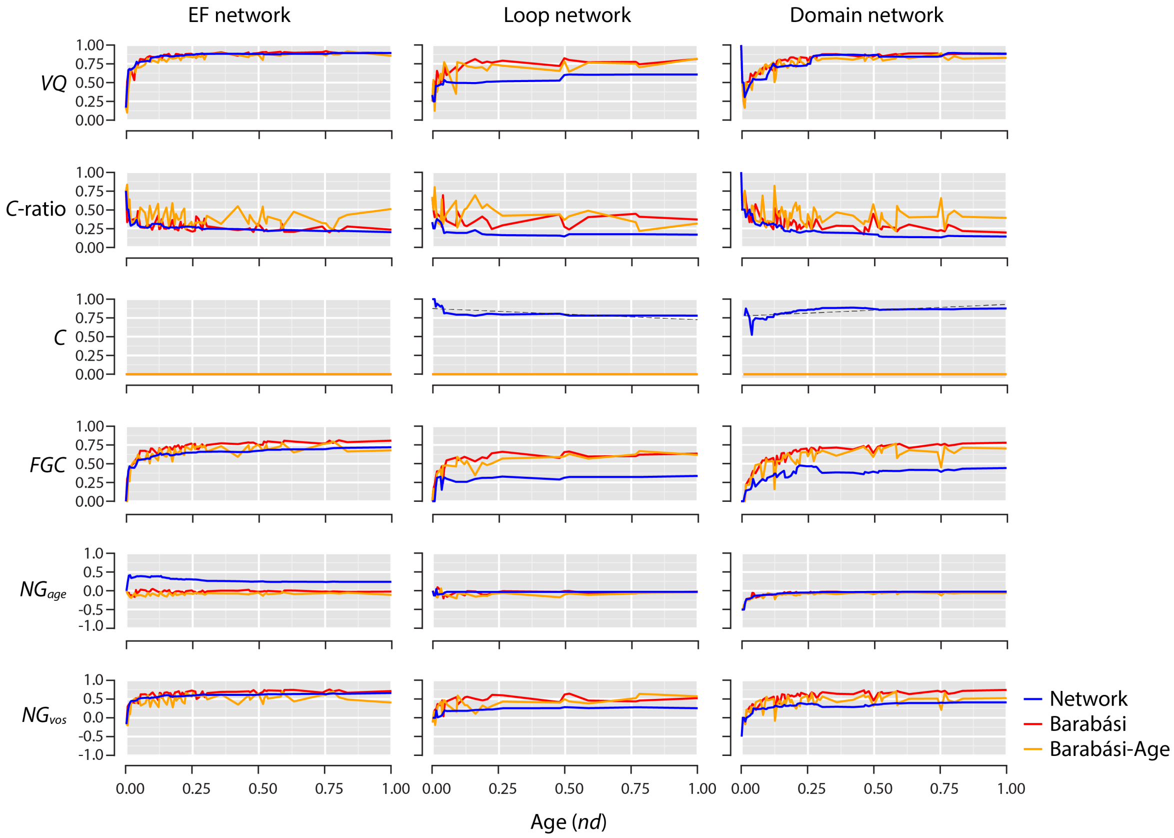

Supplementary Figure S6. Modularity indices of the EF bipartite network and its loop and domain network projections. Six indicators of modularity were plotted for each network. The coefficients of linear regression lines (grey) over *C* for the domain network were 0.0022 by network size (*N*) and 0.17 by age, and those for the loop network were -0.006 by *N* and -0.15 by age. Determination coefficients (R^2) were 0.542, 0.369, 0.802 and 0.327, in that order. The linear models shown are by *nd*. Two modeled control sets, Barabási (red) and Barabási-Age (orange) were included as reference. The "Age" control enriches preferential attachment properties in older (smaller *nd*) nodes, inducing slightly different modularity curves. Single comprehensive modularity curves were computed for each network. Modularity calculations required cumulative and weighted connectivity input. Age (*nd*) is indicated on a relative 0-to-1 scale.

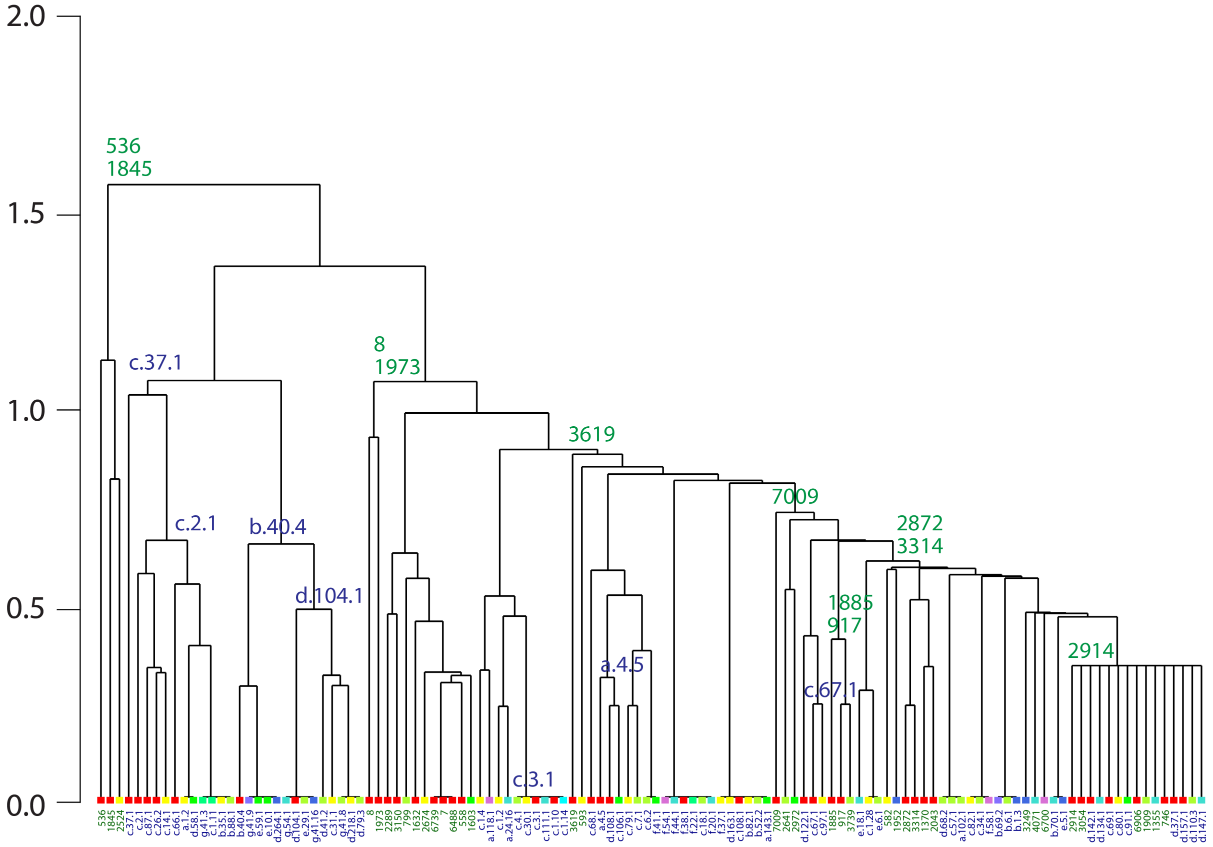

Supplementary Figure S7. Hierarchical organization of the fully-grown (extant) EF network (*nd*=1.000). NG_{age} scaled modularity matrix[39] of the last EF network in the timeline was input to calculate Euclidean distance matrix[54], which was hierarchically clustered with the Ward's minimum variance method[55]. Vertical axis represents dissimilarity between clusters of the dendrogram. Horizontal axis labels are color-coded to distinctively identify the rearrangements of the two classes of constructs. The nodes are age-sorted ascendingly within clusters, color-coded by node age and labelled using standard SCOP nomenclature[17]. The most ancient loop motifs and domain structures of the clusters comprising significant portions of the two major waves of functional innovation and the hidden EF switch of power law and modularity exchange are displayed on the roots of clades.

Supplementary Videos

Supplementary Video 1. Simulation of pairwise NG_{age} modularity of the EF network through progression of heat maps over the timeline, May 26, 2015.

Supplementary Video 2. Simulation of hierarchical organization of the EF network based on NG_{age} modularity through progression of dendrograms over the timeline, May 26, 2015.

Supplementary materials and methods

Network data analyses. The EF network and the loop and domain network projections were visualized and analyzed using Pajek[48] as they unfolded in the evolutionary timeline. The nodes (vertices) and links (arcs/edges) of the EF bipartite graph and its projections were encoded in Excel (ASCII) project files for network visualization. The quantitative network property of cumulative weighted degree per node (*cwdn*) was generated using custom Pajek macros. *cwdn* was compiled as three types of data matrices for each network (BOX 1). The data rows of matrices defined loops and domains, and were sorted in ascending order of node-(individual *nd*) or network-(event *nd*) age. Separate matrices were organized for the 'in' and 'out' degree types as well as each portion of the bipartite node set.

BOX 1. Types of data matrices

Matrix type	Matrix objective
By 'node age' (NOA)	Used for box plots and x-y line plots. Categorical columns were ordinal number, age bin, age and node label of loop and domain nodes in the networks. Additional columns described *cwdn* in increasing order of events. Rows were sorted by node.
By 'network age' (NEA)	Used for power law distribution graphs. They are essentially transpositions of NOA data. Columns were ordinal number, age bin, and age of networks, followed by *cwdn* arranged by nodes. Rows were sorted by events.
By 'degree dispersion' (DD)	Used for stacked bar charts. Column and row order are the same as NOA data. However, columns provided the distributions of final *cwdn* (i.e. at *nd*=1) across connected node age bins.

Network visualization. A set of Pajek menu commands was used to generate visualization attributes and layouts (BOX 2). Node categories were made distinguishable using various shape and color options. Evolutionary patterns were unfolded by color-coding the age of loop and domain nodes. A color palette was used that ranged from red for the most ancient node ($nd = 0$) to orchid for the most recent node ($nd = 1$).

BOX 2. Network visualization tools and commands

Pajek tool and command	Output
Network: Create Vector: Centrality: Weighted Degree: 'All', 'Output\Input'	The weighted degree vectors for undirected and directed networks, respectively.
Draw: 'Network + Vector'	Visualization of the weighted degree of nodes as node size.
Network: Create Partition: Communities: VOS clustering: 'Multi-level coarsening + Multi-level refinement'	The community-based layout of the networks, using the VOS clustering method [24, 25]. In addition, modularity indices were obtained. Default parameters were used.
Draw: 'Network + Partition' and Draw: Layers: 'In y Direction'	Vertical arrangement of nodes according to their age in both bipartite and waterfall layouts of the networks with age mappings.
Draw: Layout: Energy: Kamada-Kawai: 'Optimize inside clusters only'	Visual distribution of partitions[26] (clusters or communities) in waterfall layout, manually separated to refine the most energetically favored network configurations.

Charts and graphs. Graphing code constructs and packages of R[50,51] were used to visualize the *cwdn* (BOX 3).

BOX 3. Graphing and charting constructs and operations

R *package*: function	Result
Reshape: *	Transform textual data tables to vector form.
Lattice: *	Generate panels of network- and node-age bins.
LatticeExtra, grid: *	Resize panels.
LatticeExtra: '`panel.lmline` ()'	Draw linear regression model lines and equations, e.g. in *log-log* graphs.
Data.table: *	Edit data tables, e.g., to remove empty age bins.
igraph: *	Read network graph files to calculate modularity and apply the power law distribution model to calculate its fit statistics.
R base: '`bwplot` ()'	Box and whisker's plots based on NOA files, to depict measures of central tendency of *cwdn* over network age.
Lattice: '`xyplot` ()'	XY scatter and line plots based on NOA files drawing final *cwdn* attained at network age 1.
R color palette: *	The color-coding of data points by node age.
ggplot2: '`ggplot` ()', '`geom_bar` ()' and '`grid.draw` ()'	Produce degree dispersion stacked bar charts and graphical trend curves of power law behavior and modularity, based on DD, NEA data and VOS modularity report files, respectively.
ggplot2: `scale_fill_manual` ()	Color-coded stacked bars representing distribution of final *cwdn* over ages of connected node set.
ggplot2: '`layer` ()'	Stack negative valued bars in reflection.
ggplot2: '`theme` ()'	Color-coding of multiple curves of power law statistics and modularity indices.
gridExtra: '`grid.arrange` ()' and '`arrangeGrob` ()'	Plotting together of curves for comparison.
ggplot2: '`geom_smooth` ()'	Addition of linear regression lines to some modularity plots.
ggplot2: '`geom_tile` ()', '`scale_fill_gradient` ()', '`scale_x/y_discrete` ()' etc.	Customized modularity heat maps.
ggdendro: *	Plotting of dendrograms.
R base: '`aggregate` ()'	Calculate averages of categorized data.
plotrix: '`std.error` ()'	Measure dispersion of data through standard error.

Barabási reference networks. Reference networks for comparative analysis of power law and modularity were generated using 'barabasi'[52] methods of the R's *igraph* package[49]. The ideal power law model was implemented with '`barabasi.game` ()'. An extended model was implemented with '`aging.prefatt.game` ()'. This model simulated the scale-free evolution of random graphs by altering the probability of preference of an old vertex growing multifold, exponentially with age. Networks of three sizes, 120, 82 and 38, were created to simulate reference controls for the corresponding EF, domain and loop networks, with properties of directionality directly transferred. Ages were assigned to individual nodes in incremental order to keep age proportion per event consistent with the timelines of the test networks.

Power law statistics. *P(k)* vs. *k* and *log-log* plots provided preliminary insight into the scale free behavior of a network. Power law behavior usually manifests in degree distributions exhibiting long tails (similar to *Poisson*). R libraries and operations were utilized to run the analysis (BOX 4). The distributions were color-coded. *Log P*(*k*) vs. *log k* scatter plots supplemented analysis of power law behavior.

BOX 4. R operations used in power law analysis

R *package*: function	Result
Lattice: ‘`xyplot` ()’	Plot distributions generated from NEA files.
Data.table: ‘`table` ()’	Compute *k* frequency tables for *P(k)* vs. *k* plots.
R base: ‘`length` ()’	Calculate length of degree table in order to determine *P(k)* through quotient of *k* frequencies by maximum degree.
R base : ‘`ppois`()’	Compute *Poisson* (*k*) to be plotted against *k.*
R base: ‘`log` ()’	Implement *log P(k)* vs. *log k* scatter plots.
Lattice: ‘`lm` ()’ and ‘`panel.abline` ()’	Generate and draw linear regression models.
R base: ‘`summary (lm)$ coefficients`’	Retrieve the γ-slope power law coefficient and R^2 determination coefficient.
igraph: ‘`power.law.fit` ()’	Obtain additional statistics supporting the preferential attachment principle.

Modularity indices. Modularity indices were calculated for each network using various capabilities of the *igraph* package[49] (BOX 5). Isolated vertices were deleted using a custom function and corresponding partitions were adjusted, as required by the modularity algorithms. We borrowed the single arc of the FSF network at *nd* ~ 0.0045 to make the network at *nd* = 0 `non-empty`. *cwdn* was used as input in calculation of all modularity indices. VOS modularity indices and partitions were generated using Pajek[24,25]. *NG* modularity indices[39] were computed using two types of memberships (or partitions) as input: VOS clustering (NG_{vos}) and age (NG_{age}). Clustering ratios were determined using custom functions by dividing the number of clusters from the connected node set with the size of the connected node set.

BOX 5. R’s *igraph* package operations used in modularity analysis

igraph’s function	Result
‘`read.graph` ()’	Import network graph data.
‘`fastgreedy.community` ()’	Calculate the *FGC* modularity index [47].
‘`as.undirected` ()’	Collapse directed edges for *FGC.*
‘`modularity` ()’	Compute *NG* modularity indices.
‘`transitivity` ()’ with the ‘`average`’ option	Determine average clustering coefficient (*C*) [37, 42, 43].

Heatmaps and dendrograms. *NG* pairwise modularity matrix was computed for each network using ‘`mod.matrix` ()’[39]. Four types of memberships (or partitions) were used as input: VOS, age, *FGC* and Walk Trap Community detection (*WTC*). *WTC* is similar to *FGC* but computes communities using random walks[53]. *FGC* and *WTC* partitions were extracted by ‘`membership` ()’. Each matrix was scaled by the absolute value of the overall modularity score of the network, before being drawn as a heatmap. Pairwise scores were adjusted to -1 to make the range [-1, 1]. The x-y axis node labels were color-coded and ordered by age. Dendrograms were generated using ‘`dist` ()’ by calculating squared Euclidean distance matrices that indicate dissimilarities between the cluster means[54]. The distance (or dissimilarity) matrices were hierarchically clustered using ‘`hclust` ()’ with the Ward's minimum variance method aiming at finding compact, spherical clusters[55]. Nodes that were clustered in the dendrograms were ordered within clusters by age and were color-coded.

External support: Additional information, including scripts and workflows for visualization and statistical analyses can be found in the Molecular Ancestry Networks (MANET) database repository (http://manet.illinois.edu).
